# Can routine data be used to support cancer clinical trials? A historical baseline on which to build: retrospective linkage of data from the TACT (CRUK 01/001) breast cancer trial and the National Cancer Data Repository

**DOI:** 10.1186/s13063-017-2308-6

**Published:** 2017-11-23

**Authors:** Lucy Suzanne Kilburn, Maria Aresu, Jane Banerji, Peter Barrett-Lee, Paul Ellis, Judith Margaret Bliss

**Affiliations:** 1 ICR Clinical Trials and Statistics Unit (ICR-CTSU), Division of Clinical Studies, The Institute of Cancer Research, Sir Richard Doll Building, Cotswold Road, SM2 5NG London, UK; 2 Epidemiology and Biostatistics, School of Public Health, Imperial College London, London, UK; 3 Velindre NHS Trust Cancer Centre, Cardiff, UK; 4 Guy’s Hospital, Kings Health Partners AHSC, London, UK

**Keywords:** Randomised controlled trial, Cancer trials, Routine data linkage, Validation

## Abstract

**Background:**

Randomised clinical trials (RCTs) are the gold standard for evaluating new cancer treatments. They are, however, expensive to conduct, particularly where long-term follow-up of participants is required. Tracking participants via routine datasets could provide a cost-effective alternative for ascertaining follow-up information required to evaluate disease outcomes. This project explores the potential for routine data to inform cancer trials, using, the historical National Cancer Data Repository (NCDR) for English NHS sites and, for validation, mature data available from the TACT trial.

**Methods:**

Datasets were matched using patients’ NHS number, date of birth (dob) and name/initials. Demographics, clinical characteristics and outcomes were assessed for agreement and completeness. Overall survival was compared between NCDR and TACT.

**Results:**

A total of 3151 patients underwent linkage; 3047 (96.7%) of which had matched records. Extensive cleaning was required for some registry data fields, e.g. cause of death, whilst others had large amounts of missing data, e.g. tumour size (22.1%). Other data had high levels of matching such as dob (99.6%) and date of death (89.6%). There was no evidence of differential survival rates (8-year survival: TACT = 75% (95% CI 73, 76); NCDR = 76% (95% CI 74, 77)).

**Conclusions:**

Data quality and completeness requires improvement before routine data could be used for RCTs. Introduction of new routine datasets, including COSD, is welcomed although reporting of disease-recurrence events remains a concern. Prospective validation of such datasets is required before RCTs can confidently switch patient follow-up to utilise routinely collected NHS-based data.

**TACT Trial registration:**

Clinicaltrials.gov NCT00033683, registered on 9 April 2002; ISRCTN79718493, registered on 1 July 2001.

## Background

Randomised clinical trials (RCTs) form the gold standard methodology to determine the efficacy and effectiveness of new medical treatments. The primary endpoint in the majority of phase III trials in early breast cancer, disease-free survival, is typically analysed after a follow-up of approximately 5 years from randomisation although patients remain at risk of disease relapse and subsequent death from disease for many years thereafter. Unfortunately, partly due to cost and resource implications, there is an increased tendency to curtail follow-up beyond 5 years once the principal objective has been achieved [1]. However, if clinical trial follow-up does not continue beyond 5 years late recurrences and toxicities would not become apparent especially in patients surviving beyond 10–15 years. Meta-analyses of thousands of clinical trial patients by the Early Breast Cancer Trialists’ Collaborative Group (EBCTCG) have shown that disease-related events continue to occur out to at least 15 years [2, 3]. Thus terminating follow-up after 5 years potentially provides an incomplete understanding of treatment benefits and risks may go undetected in these otherwise healthy individuals.

Traditionally, clinical trial data collection requires each participating hospital to obtain data, retrieve missing information and forward to the coordinating clinical trials unit for analysis. The entire process can be time-consuming and labour-intensive both for hospital staff and the clinical trials units. In addition, it requires patients to either continue returning to hospitals many years after their treatment has been completed or to be reminded of their cancer diagnosis by telephone call from a research nurse [1].

Routine sources of data capture are the information regarding individual patients’ cancer diagnosis, treatment and outcomes collected by individual hospitals. These sources include data submitted by National Health Service (NHS) providers and Healthcare Quality Improvement Partnership (HQIP) commissioned national cancer audits. These data sources have not been designed specifically for clinical trials use, however, may provide a potentially cost-effective alternative to hospital-based clinical trial follow-up. In England, the National Cancer Intelligence Network (NCIN), established in 2007 and now part of Public Health England’s National Cancer Registration and Analysis Service [4], developed the National Cancer Data Repository (NCDR). At the time, this retrospectively combined data from eight English Cancer Registries, Office for National Statistics mortality data and the Hospital Episodes Statistics (HES) dataset. However, before changing clinical trial follow-up procedures, trialists need confidence that the data provided by routine sources captures the information required to answer the research question and that the datasets are of sufficient quality and completeness to replace current data collection practices. As a first step, it is helpful to establish the baseline data linkage capabilities using routine data as historically collected to understand how new and revised versions of these datasets can be structured to provide the necessary added-value to allow the switch to more efficient data capture. To do this, a project, initiated on behalf of the NCRI Breast Clinical Studies Group, to retrospectively link mature follow-up data from the Taxotere and Adjuvant ChemoTherapy (TACT) randomised clinical trial in women with early breast cancer [5, 6] with the NCDR was conducted to assess the baseline viability of linkage with routine datasets.

## Methods

**Fig. 1:**
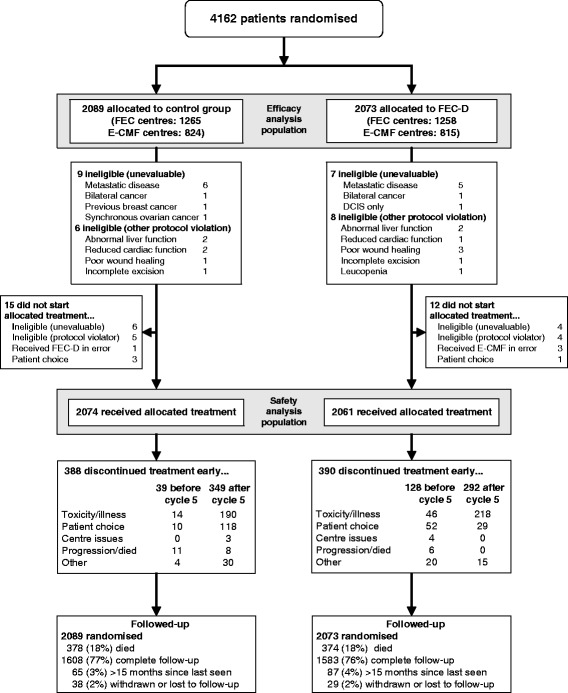
CONSORT diagram for the publication of the principal results of the TACT trial [6]. *E-CMF* epirubicin – cyclophosphamide, methotrexate, fluorouracil, *FEC-T* fluorouracil, epirubicin, cyclophosphamide – taxotere, *TACT* Taxotere and Adjuvant ChemoTherapy

**Fig. 2:**
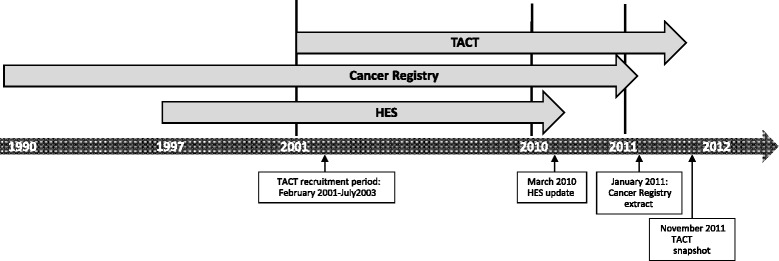
Time period of datasets. *HES* Hospital Episode Statistics, *TACT* Taxotere and Adjuvant ChemoTherapy

The UK TACT trial (CRUK01/001) randomly assigned 4162 women with node-positive or high-risk node-negative early breast cancer to sequential docetaxel after anthracycline chemotherapy [Fluorouracil, epirubicin, cyclophosphamide - taxotere (FEC-T)] or standard anthracycline chemotherapy of similar duration [FEC or epirubicin – cyclophosphamide, methotrexate, fluorouracil (E-CMF)] between February 2001 and July 2003 [5, 6]. (Consort diagram for the publication of the principal TACT results is shown in Fig. 1). Data from the 3151 TACT patients from English centres were retrospectively linked to the NCDR, containing details of cancer diagnoses, demographic information, in-patient and day-case episodes, diagnoses and operations. The linkage was based on a snapshot of TACT data extracted on 25 November 2011 when median follow-up in the trial database for all patients was 97.5 months interquartile range (IQR) (87.6; 107.4); the NCDR includes registry data up to January 2011 and HES data to March 2010 (Fig. 2). TACT patients had provided informed consent for access to routine medical records; therefore, no additional approval was required to receive data from NCIN.

TACT patients were matched to the routine datasets using their unique NHS number. The initial match was then confirmed using date of birth. Minor errors in birth records (different day or month) were ignored if the patient’s name (routinely collected in TACT) matched. Date of surgery as reported in TACT was then used to define when a patient comes under observation in the NCDR data. HES data presented multiple observations per patient depending the number of hospital outpatient/inpatient visits. A similar issue was found in cancer registry data with the same patient presenting at least two different diagnosis dates. An important stage was then the identification of a unique observation per patient. The general rule to identify the most appropriate patient/observation was to consider for each patient the nearest observation to the date of surgery provided in TACT. The record closely matching these data was retained and included in the final NCDR dataset to produce one record per patient. The key variables to identify the correct record were agreed by the project team as the date of diagnosis in the cancer registry dataset and date of hospital admission in HES. Data cleaning and standardisation of data across the data sources was undertaken before information was used for further analysis. This included validation checks, error detection and correction (e.g. patient identifiers mismatching, incorrect classifications (e.g. male)) investigation of outliers, incomplete and incorrectly formatted data (e.g. inconsistent date formatting, date of operation not always available) and establishing a consistent coding system for key variables (e.g. nodal status, tumour size (millimeter not centimetre)). Data cleaning was conducted objectively where possible (e.g. date formatting), otherwise resolution of individual issues were achieved by consensus of the project team. The level of agreement between NCDR/HES and TACT for key fields (date of death, cause of death and patient’s clinical status) was reported and overall survival and survival rates were compared using standard survival analysis methods (e.g. Cox proportional hazards model).

As distinct recording of breast cancer recurrence was not available in the NCDR, an attempt was made to see if the recurrences recorded in TACT dataset could be matched with a suitable “proxy” event in NCDR. It is known that a patient will only have oncological intervention (e.g. chemotherapy, surgery) following their primary treatment if there is evidence of recurrence and therefore the project team agreed that any oncological intervention approximately 1 year after primary treatment has been completed was to be explored as a potential indicator of recurrence. In addition, an exploratory multivariable logistic regression model was developed to see if there were any factors that predicted agreement between the TACT and NCDR datasets for distant recurrence. A forward stepwise selection method was used to select variables for inclusion within the model. Variables were included if they were statistically significant at the 1% level. The candidate covariates were year of relapse, site of relapse and centre. For modelling of centre-only data from centres with > 10 patients were included.

All analyses were conducted using Stata (version 12.1) (StataCorp, College Station, TX, USA).

## Results

**Fig. 3:**
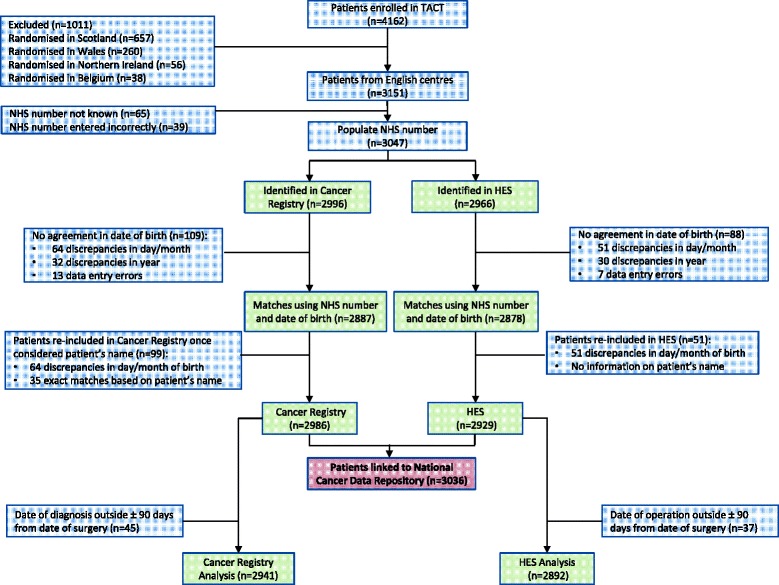
Success of linkage between TACT dataset and NCDR extraction. *HES* Hospital Episode Statistics, *NHS* National Health Service, *TACT* Taxotere and Adjuvant ChemoTherapy

Record linkage was high with 3047 (96.7%) patients successfully identified in NCDR and/or HES using the NHS number. Inevitably, when confirmation of patients’ records using birth date and name, and identification of the observation period to produce one row per patient was conducted, the success rate was slightly lower (3036 patients, 96.4%) (Fig. 3).

Level of agreement between TACT dataset and NCDR was excellent for demographic data. Staging data at diagnosis, e.g. nodal status and tumour size, showed poor concordance, primarily as a result of missing data (Table 1). Other reasons for discordance were potentially attributable to measurement unit errors (i.e. millimetre versus centimetre in recording tumour size) and likely misunderstanding of the data collection requirements and/or patient notes for certain data items by the data entry clerk at site, for example confusion between the number of nodes involved versus number of nodes examined; the latter, perhaps supported by the fact that another clinical detail, laterality of the tumour, was very well recorded. However, when considering if the level of discordance in the staging data changed clinical risk stratification, the impact was minimal with 20/1665 (1.2%) patients’ nodal status changing from positive to negative or vice versa and 31/2286 (1.4%) patients’ tumour size changing from ≤ 2 cm to > 5 cm or from > 5 cm to ≤ 2 cm.

**Table 1:** Agreement between TACT dataset and NCDR extraction

Variables	NCDR component dataset	Agreement, n (%)	No Agreement, n (%)	Missing, n (%)
Date of birth, (*n* = 3047)	Cancer Registry and HES	3036 (99.6)	11 (0.4)	–
Date of surgery^a^, (*n* = 2929)	HES	2892 (98.7)	37 (1.3)	–
Date of death^b^, (*n* = 739)	Cancer Registry	662 (89.6)	59 (8.0)	18 (2.4)
Nodal status^c^, (*n* = 2941)	Cancer Registry	1709 (58.0)	26 (1.0)	1206 (41.0)
Nodes involved^c^, (*n* = 2941)	Cancer Registry	1581 (53.8)	85 (2.8)	1275 (43.3)
Nodes examined^c^, (*n* = 2041)	Cancer Registry	1469 (50.0)	229 (7.8)	1238 (42.2)
Tumour size^c^, (*n* = 2941)	Cancer Registry	2075 (70.6)	211 (7.2)	651 (22.1)
Side of tumour^c^, (*n* = 2941)	Cancer Registry	2850 (96.9)	46 (1.6)	44 (1.5)
Tumour grade^c^, (*n* = 2941)	Cancer Registry	2181 (74.5)	668 (22.8)	79 (2.7)

*TACT* Taxotere and Adjuvant ChemoTherapy, *NCDR* National Cancer Data Repository, *HES* Hospital Episode Statistics

^a^Matches using NHS number, date of birth and patient’s name and surname

^b^Denominator = number of deaths in TACT once linked to the Cancer Registry

^c^Matches using NHS number, date of birth and patient’s name. Clinical status assessed within 90 days from date of surgery as entered in TACT

**Fig. 4:**
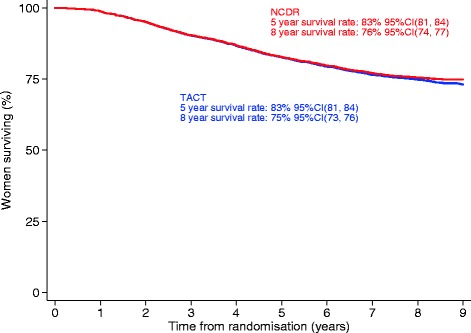
Difference in overall survival between TACT dataset and NCDR extraction (*red* = NCDR data, *blue* = TACT data). *NCDR* National Cancer Data Repository, *TACT* Taxotere and Adjuvant ChemoTherapy

Date of death was recorded for 748 patients in TACT dataset; nine deaths occurred after the last NCDR data extract and therefore were censored. Eighteen patients who had died according to the TACT dataset had no death recorded in the NCDR. In addition, the Cancer Registry contained an extra 27 death notifications that were not available in the TACT dataset. Despite these discrepancies, there was no evidence of a difference in overall survival between the two datasets (Fig. 4). Eight-year survival rates were 75% (95% CI 73, 76) in TACT dataset and 76% (95% CI 74, 77) in NCDR, respectively.

Of the 2929 TACT patients who were correctly identified in NCDR and for whom HES data was available, 898 (30.7%) had had a disease-free survival (DFS) event (140 local recurrence, 691 distant recurrence or 67 new breast second primary cancer) reported in the TACT dataset. Any DFS event in TACT dataset after the reporting date of the HES dataset was noted but not considered a discrepancy case (2 local, 22 distant recurrences and 1 second primary cancer). The level of matching between a DFS event in TACT dataset and a hospital episode suggesting relapse reported in NCDR was 71.0% (98 events) for local recurrence, 63.5% (425 events) for distant recurrence and 81.8% (54 events) for new breast second primary cancer (Table 2), precluding survival analysis based verification of the trial’s primary endpoint. For distant recurrences reported, a multivariable model suggested that distant recurrences were more likely to be identified in the NCDR dataset if they included metastases to the brain. Conversely, disagreement was more likely if the relapse was recent or if the patient had lung metastases. Degree of agreement also varied between centres (Table 3).

**Table 2:** Identification of TACT-reported disease-free survival events in NCDR

	Local recurrence	Distant recurrence	New breast disease
	(*N* = 140)	(*N* = 691)	(*N* = 67)
Agreement^a^	98 (71.0%)	425 (63.5%)	54 (81.8%)
Event reported in TACT but not in NCDR	17	76	2
Disagreement in number of sites and/or diagnosis time	23	168	10
Event occurred after 31 March 2010 (HES extract date)	2	22	1

^a^Denominator excludes events occurring after 31 March 2010 (HES extract date). *TACT* Taxotere and Adjuvant ChemoTherapy, *NCDR* National Cancer Data Repository, *HES* Hospital Episode Statistics

**Table 3:** Logistic regression model identifying factors for distant recurrence agreement between TACT and routine datasets

	Odds ratio	95% CI	*P* value
Year relapse reported	0.88	0.80, 0.97	0.01
Distant relapse site = lung	0.61	0.38, 0.98	0.04
Distant relapse site = brain	2.96	1.29, 6.77	0.01
Centre [A]	0.28	0.07, 1.09	0.07
Centre [B]	0.41	0.06, 2.98	0.38
Centre [C]	1.31	0.19, 8.83	0.78
Centre [D]	0.18	0.03, 0.99	0.05
Centre [E]	0.29	0.05, 1.58	0.15
Centre [F]	0.30	0.06, 1.45	0.14
Centre [G]	0.34	0.06, 1.83	0.21
Centre [H]	0.04	0.01, 0.23	< 0.001
Centre [I]	0.05	0.01, 0.29	0.001
Centre [J]	0.07	0.02, 0.33	0.001
Centre [K]	0.28	0.05, 1.52	0.14
Centre [L]	0.31	0.05, 1.92	0.21
Centre [M]	0.37	0.06, 2.29	0.29
Centre [N]	0.37	0.09, 1.61	0.19
Centre [O]	0.07	0.01, 0.43	0.004
Centre [P]	0.23	0.05, 1.19	0.08
Centre [Q]	0.66	0.09, 4.60	0.68
Centre [R]	0.06	0.01, 0.37	0.002
Centre [S]	0.14	0.03, 0.58	0.007
Centre [T]	0.19	0.04, 0.84	0.03
Centre [U]	0.11	0.02, 0.60	0.01
Centre [V]	0.08	0.02, 0.39	0.002
Centre [W]	0.15	0.04, 0.60	0.007
Centre [X]	0.07	0.01, 0.38	0.002
Centre [Y]	0.10	0.02, 0.50	0.005

OR < 1 imply distant recurrence less likely to be identified in NCDR dataset compared to gold standard TACT dataset. Individual centres included in the model have been anonymised. *TACT* Taxotere and Adjuvant ChemoTherapy

## Discussion

The retrospective linkage of the TACT dataset and NCDR has shown that where data exist, routine data is of reliable quality, i.e. agreement > 70% for the majority of variables matched. Some of the issues related to using traditional routine datasets include inherent biases such as the amount of missing data, in particular, staging and recurrence details and also the time lag in receiving data. This can result in informative censoring. Within the context of this project we are matching within a pre-defined population of trial patients and not attempting to extrapolate to the general population to estimate incidence rate thus some of the biases known to occur in routine datasets may be less problematic for use within clinical trials.

In relation to data characterisation, centres may also benefit from further guidance to avoid misunderstanding of data entry requirements. Lack of standardisation across registries was also problematic requiring the data to be cleaned prior to starting any analysis; the most time consuming part of this validation exercise. Examples include data recorded in the wrong fields, e.g. clinical stage recorded as pathological stage, and inconsistent data formats within a field (coding versus free text). However, once cleaned, comparisons of overall survival, one of the key TACT endpoints, show similar conclusions are drawn from basic outcome data whether using routine data for follow-up versus traditional data collection methods. Little can be said about cancer recurrence, which is often the primary endpoint of phase III cancer RCTs, as data were not consistently available in NCDR at the time and therefore is an unfair comparison. However, a 70% ascertainment rate for an endpoint would not under other circumstances be considered acceptable for a clinical trial, and whilst no evidence of bias was observed, it would be difficult to rule out. Therefore, availability of properly specified recurrence data is a prerequisite for the future utility of routine datasets.

Recently, new national datasets have been introduced to improve the quantity and quality of cancer information collected including specific details of radiotherapy and systemic treatments and a new minimum core dataset – the Cancer Outcomes and Services Dataset (COSD) – capturing basic information on treatment, diagnosis and death that now includes details on cancer recurrence, a key outcome for cancer RCTs [7]. The resulting linked data aims to allow each patient’s treatment pathway to be mapped from diagnosis to cure or death with emphasis relating to collecting cancer-specific information. Given our experience to date, the new information on recurrence, e.g. date of recurrence, required to make the switch to routine viable for cancer clinical trials, should be reasonably captured in the COSD. However, site of recurrence will not be recorded so easily, therefore analyses exploring patterns of relapse and more specific recurrence-related endpoints, such as time to distant recurrence, represent a considerable limitation for its utility.

The strength of this study is that this was a large multicentre study and so routine data will have been collected from a number of different hospitals across England giving a realistic impression of the quality and variability of data available. Unfortunately, at the time of this study the newer datasets were not available. This study provides a useful baseline from which to compare the new datasets; however, the matching process using the newer datasets will be required.

Now that a baseline has been established, the next phase is to prospectively evaluate the new routine datasets with contemporary trial data. Working with the National Cancer Registration and Analysis Service (NCRAS) within Public Health England, the ICR Clinical Trials and Statistics Unit (ICR-CTSU) will help validate the COSD, the Systemic Anti-Cancer Therapy (SACT) dataset, radiotherapy dataset (RTDS) and HES using data initially from the TACT2 (ISRCTN68068041) [8], POETIC (ISRCTN63882543), IMPORT HIGH (ISRCTN47437448) and FAST FORWARD (ISRCTN19906132) breast cancer trials.

This prospective validation study will identify whether routine datasets are of sufficient standard to replace traditional data collection methods. The objectives are to identify and quantify the number of trial participants within each relevant dataset; conduct an objective assessment of routine data completeness, validity, and consistency with trial data; a cross-comparison of trial baseline and treatment data and emerging disease-related outcome data; collection of long-term safety data and identifying the representativeness of trial patients versus general population matched to the trial’s main criteria (e.g. tumour characteristics). After investigating the data from breast cancer trials, the plan is to extend beyond breast cancer into other disease areas, e.g. prostate, once the project is fully established. The aim is to improve quality and completeness of routine data via a two-way data exchange whilst also allowing a longitudinal assessment to see when these datasets may be of a sufficient standard, particularly the data relating to recurrence, to replace traditional data collection methods for RCTs.

Using routine datasets as an alternative for clinical trial follow-up data collection shows promise but the switch is unlikely to happen in the near future. Phase III clinical trials are initiated with the aim of influencing clinical practice, therefore follow-up data needs to be complete, contemporary and of high quality to ensure results are robust. Missing data is a key problem for routine data, particular of registry data, and increased standardisation would enhance accessibility. This can be achieved in part through increased training of hospital coding staff and the switch to the single shared registry system, English National Cancer Online Registration Environment (ENCORE).

As clinical trials are often run throughout the whole of the UK and beyond, another recommendation would be for the NCRAS to integrate data from the devolved nations to allow access to UK-wide registry data in one application process. Currently, this requires separate applications and it is not obvious to clinical trial researchers how to gain access to these datasets.

If routine data was adopted in a future clinical trial in the place of centre-based follow-up we would recommend that data ‘cleaning’ has an appropriate level of quality control checking as part of the trial’s standard data monitoring plan. This needs to be efficient and proportionate to the risk of the trial. For example, trials of an investigational medicinal product used in an unlicensed indication would require a higher amount of checking. Similarly, fields related directly to primary endpoint evaluation would require more validation. Data monitoring plans should be agreed by the trial team as early as possible. For routine data we recommend both “in house” verification of data as part of central statistical data monitoring whilst allowing the possibility of direct contact with centres for clarification of significant suspected systematic issues with data quality.

Using routine datasets to facilitate long-term follow-up should reduce the burden on research teams in hospitals allowing them to focus on higher-risk patients. In addition, patients should benefit by avoiding unnecessary follow-up visits. The resource saving may be less clear for clinical trials units. While switching to routine data use may reduce costs, the amount of time required to clean, process and merge routine data (with either in-house data collection or even datasets from other counties) may increase the workload substantially for the trials unit data managers and statisticians.

Finally, routine data needs to be updated frequently to be able to compete with the traditional data collection methods. The lag time for availability of registry and HES data in the NCDR dataset used for this validation study meant that the most recent data available in TACT could not be used for comparison. In addition, the time taken to request, receive and process routine data will need to be minimised to avoid delays in publishing a practice-changing result which would counterbalance the cost-effectiveness of the data collection method.

## Conclusions

The overall aim for trialists, hospitals and patients is for clinical trials to run more efficiently with a reduced resource burden and this may be achieved by using routine data sources. Using routine data sources allows centre staff to prioritise patients who require further intervention for their cancer and allows clinical trials to maximise information gathered to answer the research questions within the trial design; thus ultimately improving patient care. By working with NCRAS to prospectively validate the quality of routine data compared with traditional methods we can identify whether routine data can be used for clinical trial follow-up purposes in a more timely manner.

## Abbreviations

COSD: Cancer Outcomes and Services Dataset
DFS: Disease-free survival
DOB: Date of birth
EBCTCG: Early Breast Cancer Trialists’ Collaborative Group
E-CMF: Epirubicin – cyclophosphamide, methotrexate, fluorouracil
ENCORE: English National Cancer Online Registration Environment
FEC-T: Fluorouracil, epirubicin, cyclophosphamide - taxotere
HES: Hospital Episode Statistics
ICR-CTSU: Institute of Cancer Research Clinical Trials and Statistics Unit
IQR: Interquartile range
NCDR: National Cancer Data Repository
NCIN: National Cancer Intelligence Network
NCRAS: National Cancer Registration and Analysis Service
NCRI: National Cancer Research Institute
NHS: National Health Service
RCT: Randomised controlled trial
RTDS: Radiotherapy dataset
SACT: Systemic Anti-Cancer Therapy
TACT: Taxotere and Adjuvant ChemoTherapy
